# Ocular and Periocular Metastasis in Breast Cancer: Clinical Characteristics, Prognostic Factors and Treatment Outcome

**DOI:** 10.3390/cancers16081518

**Published:** 2024-04-16

**Authors:** Yacoub A. Yousef, Mona Mohammad, Hanan Khalil, Tala Khouri, Rand Alsweiti, Jakub Khzouz, Dima Abu Laban, Imad Jaradat, Ahmad Kh. Ibrahimi, Akram Al-Ibraheem, Mahmoud Al Masri, Ibrahim AlNawiaseh, Hikmat Abdel-Razeq

**Affiliations:** 1 Department of Surgery (Ophthalmology), King Hussein Cancer Center, Amman 11941, Jordan; dm.11804@khcc.jo (M.M.); khouri.tala13@gmail.com (T.K.); randsweiti@yahoo.com (R.A.); i-nawaiseh@khcc.jo (I.A.); 2 Department of Medical Oncology, King Hussein Cancer Center, Amman 11941, Jordan; hk.10660@khcc.jo; 3 Department of Pathology, King Hussein Cancer Center, Amman 11941, Jordan; jkhzouz@khcc.jo; 4 Department of Radiology, King Hussein Cancer Center, Amman 11941, Jordan; 5 Department of Radiation Oncology, King Hussein Cancer Center, Amman 11941, Jordan; ijaradat@khcc.jo (I.J.); aibrahimi@khcc.jo (A.K.I.); 6 Department of Nuclear Medicine, King Hussein Cancer Center, Amman 11941, Jordan; aibraheem@khcc.jo; 7 Department of Surgery, King Hussein Cancer Center, Amman 11941, Jordan; malmasri@khcc.jo; 8 School of Medicine, The University of Jordan, Amman 11942, Jordan

**Keywords:** breast cancer, ocular metastasis, periocular metastasis, choroidal metastasis, treatment outcomes

## Abstract

**Simple Summary:**

Breast cancer remains a leading cause of cancer-related mortality and morbidity worldwide. Ocular and periocular metastasis present as a rare but clinically significant manifestation. This study is crucial for understanding the rare occurrence of ocular and periocular metastasis in breast cancer. By exploring demographics and clinical aspects, we aim to improve the management of this condition. Our focus is on enhancing treatment strategies, predicting outcomes, and ultimately improving the quality of life for breast cancer patients with ocular metastasis. This study addresses the gaps in knowledge regarding the intricacies of this manifestation, driving progress toward more effective interventions and better patient outcomes.

**Abstract:**

Background: Breast cancer remains a leading cause of cancer-related mortality and morbidity worldwide. Ocular and periocular metastasis present as a rare but clinically significant manifestation. This study aims to explore demographics and clinical aspects of ocular and periocular metastasis in breast cancer patients. Methods: A retrospective cohort study comprising 45 breast cancer patients with ocular or periocular metastasis treated between 2013 and 2023. Patient demographics, tumor characteristics, diagnostic methods, treatment modalities, visual outcomes, and survival data were analyzed. Results: Among 9902 breast cancer patients, 0.5% developed ocular or periocular metastasis, constituting 2.4% of metastatic cases. The median age was 50 years. Ocular metastasis timing varied: 5% before breast cancer, 24% concurrent, 22% within a year, and 49% after. The most common presentations included incidental MRI findings (42%) and vision decline (31%). Metastasis involved the orbit (47%), choroid (40%), optic nerve (11%), and iris (2%), with 44% having bilateral involvement. Predictive factors included invasive lobular carcinoma (ILC) (*p* < 0.0001) and brain metastasis (*p* < 0.0001), with ILC exhibiting a sixfold higher likelihood of ocular metastasis than invasive ductal carcinoma (IDC). Primary treatment was radiation therapy (89%), yielding a 55% maintenance of excellent vision (<0.5), with 93% developing dry eye disease. Patients with ocular metastasis faced an increased risk of disease-related mortality (*p* < 0.0001), with 71% succumbing within 10 months post-diagnosis. Conclusions: Ocular metastasis in breast cancer is rare (0.5%) but signifies poor outcome. It is linked to ILC and concurrent brain metastasis. Primary treatment involves radiation therapy, with a favorable visual prognosis.

## 1. Introduction

Breast cancer stands out as the prevailing malignancy globally and within the Eastern Mediterranean Region, serving as the primary cause of cancer-related fatalities among women [1,2]. In 2020 alone, almost 2.3 million new cases of breast cancer emerged worldwide, resulting in 685,000 fatalities [1,3]. In Jordan, cancer ranks as the second principal cause of mortality following cardiovascular diseases, with breast cancer accounting for the third highest number of cancer-related deaths, trailing behind lung and colorectal cancers [3,4]. 

Breast cancer, a leading cause of morbidity and mortality among women, is characterized by its propensity to metastasize to distant sites [1]. While the majority of metastases involve predictable organs such as bones, lungs, and liver, breast cancer can manifest atypical metastatic patterns, including rare occurrences in the ocular and periocular regions [5]. Ocular metastasis from breast cancer remains a clinical enigma, demanding heightened awareness for timely recognition and optimal management. The incidence of ocular and periocular metastasis in breast cancer, though relatively low at 0.5% [6], represents a critical facet of the disease’s heterogeneity. 

Ocular metastasis represents an infrequent occurrence among cancer patients, with breast cancer accounting for the highest proportion of primary sites (28.5–58.8%) [7,8,9,10]. The increasing prevalence of ocular metastases originating from breast cancer can be attributed to recent advancements in systemic breast cancer treatment, leading to prolonged survival and enhanced diagnostic capabilities [8,9]. Typically, ocular metastases coincide with the systemic progression of previously identified breast cancer. However, in 25% of cases, ocular metastases are identified in patients with de novo breast cancer as an initial manifestation [10].

This study aims to explore demographics, clinical aspects, diagnosis, and management of ocular and periocular metastasis in breast cancer patients. Understanding the peculiarities of ocular metastasis is paramount in the context of breast cancer management. Ocular involvement can substantially affect patients’ visual function and overall quality of life, necessitating an adequate approach to diagnosis, treatment, and follow-up care. As the literature on this specific aspect is limited, this study endeavors to contribute valuable insights to enhance the comprehension and clinical management of ocular metastasis in breast cancer.

## 2. Materials and Methods

This is a retrospective cohort study of 45 patients with breast cancer who were treated at the King Hussein Cancer Center (KHCC) from 2013 to 2023 and had a clinical and/or pathological diagnosis of ocular or periocular metastasis. Study inclusion required access to patient medical records and data registry. The data collected included patient age, sex, laterality, age at diagnosis of breast cancer, pathology of breast cancer, site of ocular metastasis, method of diagnosis of ocular metastasis, treatment of the ocular metastasis, visual outcome, and survival. The KHCC Institutional Review Board approved this study (23KHCC146); given the retrospective nature of our study and lack of any identifier, consent was waived.

### 2.1. Inclusion and Exclusion Criteria

The eligibility criteria for inclusion comprised patients with pathological diagnosis of breast cancer who developed or presented with intraocular metastatic breast cancer or orbital soft tissue metastasis including the lacrimal gland, extraocular muscles, optic nerve, and orbital fat. Metastasis of an origin other than breast cancer was excluded, and metastasis to the orbital bone or cavernous sinus was not considered ocular or periocular metastasis.

### 2.2. Ocular Metastasis: Characteristics, Definitions, and Treatment Approaches

All the patients included in this study had a confirmed pathologic diagnosis of breast cancer, mostly invasive ductal or invasive lobular types. The metastasis was divided into ocular metastasis (including intraocular structures such as the choroid, the optic nerve head, and the iris) and periocular metastasis (including the lacrimal gland, the extraocular muscles, the orbital fat, and the orbital optic nerve). The clinical diagnosis of intraocular metastasis was based on clinical evaluation by at least one ocular oncologist. For periocular metastasis, we relied on radiological evidence from magnetic resonance imaging (MRI), which was confirmed by pathology in certain cases. 

For ocular or periocular metastasis treated by fractionated external beam radiation therapy, the standard radiation dose was 30 Gy/15 Fx/2 weeks, using 6 MV photons, via VMAT technique (Elekta, HD Versa linear accelerator) (Elekta, Atlanta, GA, USA). The chemotherapy regimen for metastatic breast cancer was highly variable depending on multiple factors like sites of metastasis, tumor activity, hormone-receptor status, and others. We evaluated vision using decibel measurements in the ocular oncology clinic at the time of diagnosing ocular metastasis and after treatment. Choroidal metastasis response was documented through color fundus photos and B-scan images.

### 2.3. Statistical Analysis

A descriptive analysis of patients’ information was conducted. Categorical data, such as age group, grade, and other factors, were presented as counts and percentages. The Kaplan–Meier method was used to estimate OS curves, *p* values were measured with Fisher’s exact tests, and values of 0.05 or less were considered significant.

## 3. Results

During the 10-year study period, a total of 9902 patients with breast cancer included in our hospital-based cancer registry were screened for ocular or periocular metastasis. Out of 1888 (19.1%) patients with metastatic breast cancer, 45 (0.5%) were found; all were female and developed ocular or periocular metastasis, constituting 2.4% of metastatic cases. Among the group with ocular and periocular metastasis, six (13%) had bilateral breast cancer, and the median age at diagnosis of breast cancer was 48 (31–71) years; 22 cases (49%) were postmenopausal and 12 (27%) had a family history of breast cancer. The median duration between diagnosis of breast cancer and ocular metastasis was 10 months (range 1–65 months). A total of 2 cases (4%) had ocular metastasis diagnosed before breast cancer, 11 (24%) were diagnosed with ocular or periocular metastasis at the same time as breast cancer, 10 (22%) were diagnosed within 1 year after breast cancer, and 22 (49%) were diagnosed after 1 year of breast cancer. Twenty-seven cases (60%) had concomitant brain metastasis.

### 3.1. Presentation and Ocular Features

**Figure 1:**
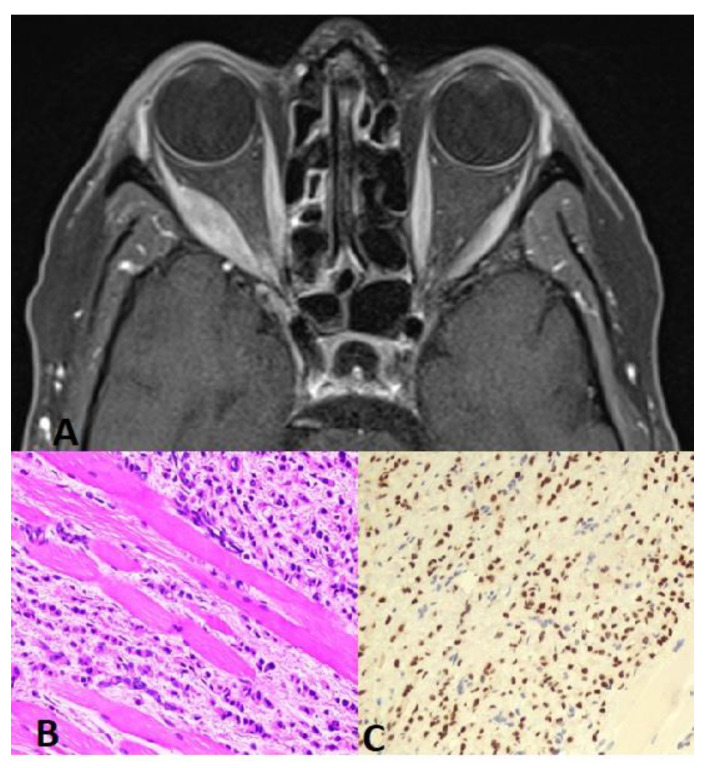
(**A**) Orbit CT axial scan shows soft tissue mass with involvement of the right lateral rectus muscle and extension into the right periorbital space. This is associated with partial encasement and displacement of the right optic nerve and mild proptosis. (**B**) Muscle biopsy: H&E 20× higher magnification reveals metastatic lobular carcinoma of the breast infiltrating the skeletal muscles. Note the linear/single-cell file arrangement of tumor cells (Indian-file pattern). (**C**) GATA3 20× tumor cells show nuclear positivity for GATA3 immunostain, confirming breast origin.

**Figure 2:**
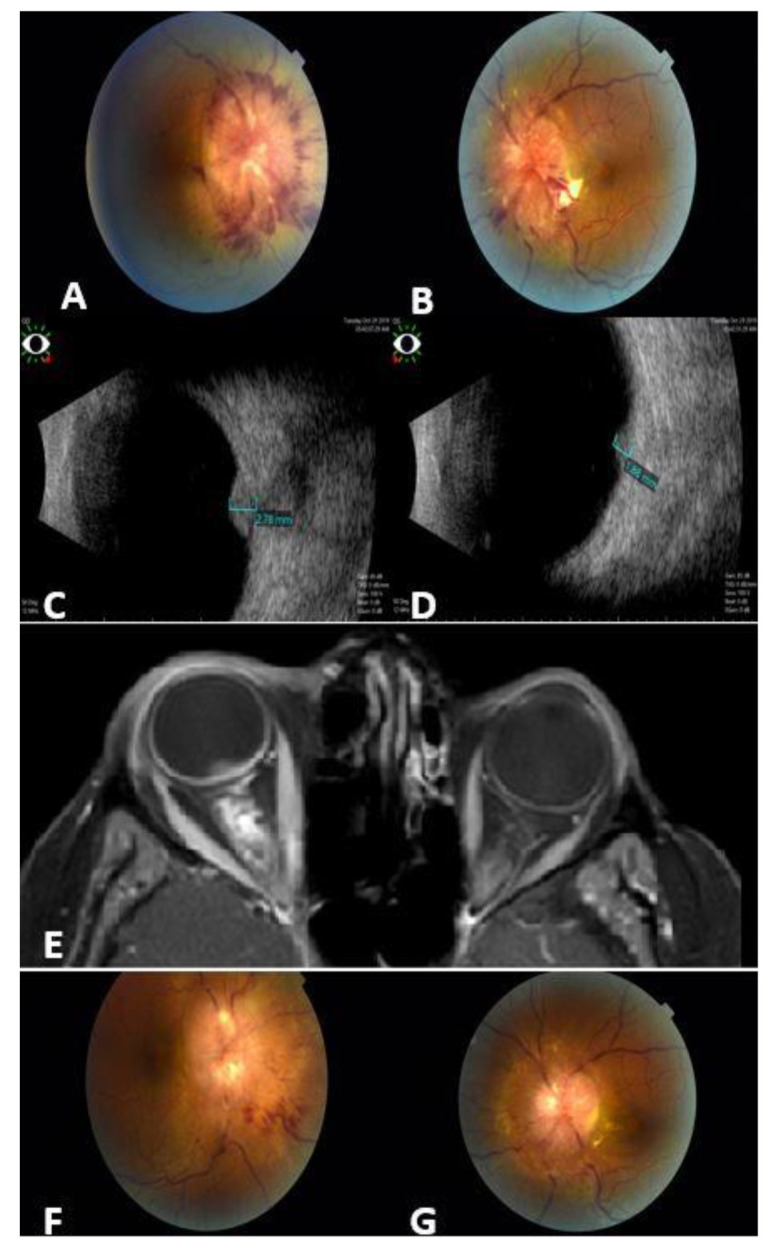
A 43-year-old female with metastatic breast cancer to the optic nerves. Fundus photos for the right (**A**) and left (**B**) eyes show bilateral infiltrates around the optic nerves, more prominent in the right eye (**A**). Ocular B-scan photos show clearly the hyperechoic masses over the right (**C**) and left (**D**) optic nerves. Orbit MRI Axial T1-weighted scan with contrast (**E**) shows thickening of the optic nerve sheath that extends to the optic nerve head (more prominent on the right side), irregular outline, and stranding of surrounding fat planes. Fundus photos for the same patient 3 months post orbit EBRT: major regression in optic nerve infiltrates in both eyes: right (**F**); left (**G**).

The median age of patients at the time of ocular metastasis diagnosis was 50 (35–79) years, and the most common way of diagnosis was incidental by MRI, performed for a different clinical indication, in 19 (42%), followed by visual complaints in 14 (31%) patients. The most common anatomic site of ocular metastasis was the orbit in 21 (47%) patients, including the extraocular muscles in 3 patients (Figure 1), followed by the choroid in 18 (40%), the optic nerve in 5 (11%) (Figure 2), and the iris in 1 case (2%). Regarding the 18 cases with choroidal metastasis, 7 (39%) had bilateral multifocal lesions, 2 (11%) had unilateral multifocal, and 9 (50%) had unilateral unifocal lesion. All the lesions were elevated plaque-like choroidal lesions with indistinct borders (white–yellow in color). No one was pink, bright orange, or brown in color. The total number of lesions was 34 lesions. In total, 30 lesions (88%) were larger than the optic disc in diameter, 29 (85%) lesions were in the posterior pole, and 31 (91%) were associated with exudative retinal detachment. All the lesions were less than 5 mm in thickness. For the 65 affected eyes, the visual acuity at diagnosis was better than 0.5 in 36 eyes (55%), between 0.1 and 0.5 in 19 eyes (29%), and worse than 0.1 in 14 eyes (22%).

Nineteen (42%) patients were asymptomatic and were found to have ocular metastasis (8 patients) or periocular metastasis (11 patients) incidentally by MRI. The final diagnosis of metastatic breast cancer to the intraocular structures (including the choroid or the iris) was always clinical, which counts for 19 (42%) patients. A total of 20 (44%) patients were diagnosed radiologically based on MRI findings (without pathology), including 5 patients with optic nerve metastasis and 15 patients with orbital metastasis. Only six (13%) patients had pathological confirmation of metastasis to the orbit (Table 1). Out of 45 cases, 20 (44%) had bilateral metastasis and, for the 18 cases with choroidal metastasis, 7 (39%) had bilateral multifocal lesions, 2 (11%) had unilateral multifocal, and 9 (50%) had unilateral unifocal lesion. Of interest, all patients with bilateral ocular metastasis had unilateral breast cancer. 

### 3.2. Prognostic Factors for Ocular Metastasis in Breast Cancer

The pathology of the primary breast cancer was invasive ductal carcinoma (IDC) in 28 cases (62%) and invasive lobular carcinoma (ILC) in 17 cases (38%). The prognostic factors for a higher chance of ocular metastasis in breast cancer include ILC and the presence of brain metastasis (*p* > 0.0001). Moreover, patients with ocular metastasis were found to have a higher risk of disease-related mortality Table 2. Patients with ILC had a 2.1% chance to present with ocular or periocular metastasis, which is six times more than IDC (0.35%). Patients with ILC were more likely to have metastasis to the optic nerve (*p* = 0.036) and more likely to have concomitant brain metastasis (*p* = 0.039); see Table 3. 

### 3.3. Management and Outcome

**Figure 3:**
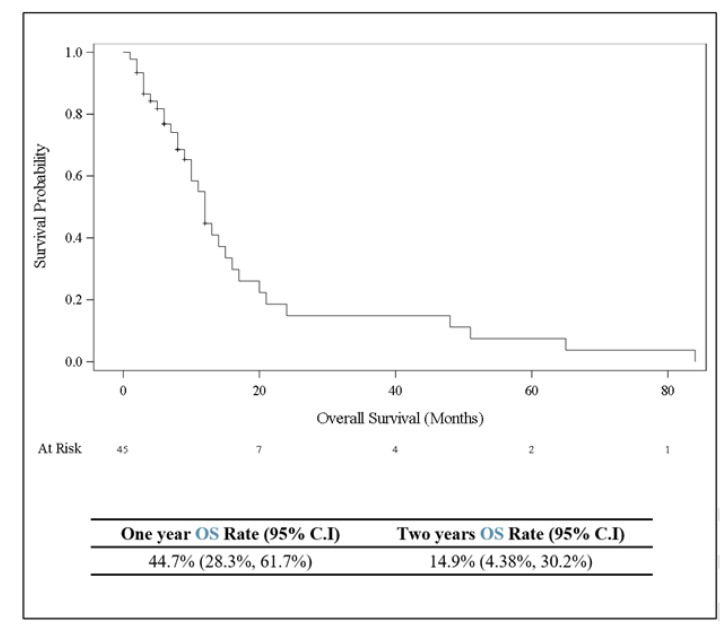
Kaplan–Meier curve showing overall survival (in months) for 45 patients with breast cancer who developed ocular or periocular metastasis.

Out of 45 patients with ocular metastatic breast cancer, 5 (11%) were treated by systemic chemotherapy and 40 (89%) were treated by radiation therapy. The standard radiation dose was 30 Gy/15 Fx (summary of outcome in Table 4). The most common ocular complications of treatment were dry eye disease in 42 cases (93%), radiation keratopathy in 4 patients (8%), radiation retinopathy in 3 (7%), cataract in 2 (4%), and periocular skin redness in 6 (13%). The visual acuity after treatment was better than 0.5 in 36 cases (55%), between 0.1 and 0.5 in 17 cases (26%), and worse than 0.1 in 12 eyes (18%). At a median follow-up of 8 months (mean 13 months, range 1–81 months) after diagnosis of ocular metastasis, no single eye showed recurrence of the ocular metastasis and, at the last date of follow-up, 32 patients (71%) were dead (Figure 3 Kaplan–Meier curve).

## 4. Discussion

Ocular and periocular metastasis from breast cancer is uncommon, and breast carcinoma is the most common primary tumor, accounting for 28.5–58.8% of all orbital metastases. This is followed by lung cancer (24%) and skin melanoma (14%) [10,11,12]. The presence of ocular and periocular metastasis in breast cancer is a rare but impactful phenomenon, necessitating a detailed examination of its clinical aspects. In our study involving 9902 breast cancer patients, over a decade, 1888 patients had metastatic breast cancer, and ocular and periocular metastasis were identified in 45 individuals, comprising 0.5% of all cases and 2.4% of metastatic cases, which is almost close to Shields et al.’s findings of ocular metastasis in 4% of metastatic breast cancer cases [13]. This relatively low frequency underscores the importance of understanding its clinical features and the need for vigilant monitoring, particularly in advanced-stage breast cancer.

Ocular metastases from breast cancer can occur at various stages of the disease. In our analysis, 4% of cases presented with ocular metastases before the patient was aware of breast cancer, 24% at the initial presentation of breast cancer, and 72% as part of the systemic progression of previously diagnosed breast cancer. The time interval between breast cancer diagnosis and ocular metastasis varies widely, ranging from 1 month to 25 years [9,10,14,15]. In our study, the median duration between breast cancer diagnosis and ocular metastasis was 10 months, ranging from 1 to 65 months, aligning with previous findings [16]. Examining the demographic profile, all patients with ocular metastatic breast cancer were females, consistent with the general distribution of breast cancer [17].

The median age at diagnosis among women in Jordan is 51 years, which is approximately 10 years younger than the median age at diagnosis of breast cancer for women in Western countries [4] and, similarly, the median age at the diagnosis of ocular metastasis in our cohort was 50 years, indicating its occurrence across different age groups. Prevalent factors included a family history of breast cancer, postmenopausal status, and advanced tumor stages, aligning with established risk factors for aggressive breast cancer phenotypes [18]. Our findings resonate with Cristofanilli et al.’s report on a higher incidence of metastatic breast cancer in postmenopausal women [19]. However, of interest, our cohort showed a higher prevalence of ocular metastasis in patients with a negative family history of breast cancer. This difference can be attributed to the low number of patients in this rare entity.

Diagnosing choroidal metastasis relies on clinical assessment, where the patient presents usually with creamy yellow choroidal lesions that can be unifocal, multifocal, or bilateral and associated with exudative retinal detachment. Diagnostic tools such as ocular ultrasonography (US) and optical coherence tomography (OCT) contribute valuable insights. B-scan US typically depicts choroidal metastases as predominantly plateau-shaped, occasionally dome-shaped, or rarely mushroom-shaped. A-scan findings often reveal high or medium reflectivity, exhibiting an internal V-shaped pattern in thicker metastases [11,20]. These lesions generally display minimal or no internal vascularization, accompanied by the presence of serous retinal detachment. OCT facilitates a detailed examination of retinal changes associated with choroidal metastases, including convex retinal profiles, serous neurosensory detachments, displacement of the photoreceptor layer due to sub-retinal fluid, alterations in inner retinal layers with hyper-reflective irregular spots on the Retinal Pigment Epithelium (RPE), and other features [11,20,21,22]. The presentation of ocular metastasis exhibited variability, with MRI frequently detecting cases incidentally, underscoring the importance of imaging studies. However, a substantial proportion manifested with a decline in vision, emphasizing the diverse clinical presentations. Recognizing these varied presentations is crucial for timely diagnosis and intervention, in line with observations by Arepalli et al., which highlight the heterogeneity in the clinical presentation of ocular metastasis [23]. Regarding the affinity of breast cancer cells to specific ocular tissues, there is controversy. Extraocular muscles are considered rare sites for metastasis [24], possibly due to constant movement preventing cell lodging [25]. However, in our cohort, the orbit was the most common site of metastasis (47%), including 7% of cases with metastasis in an extraocular muscle, followed by uveal metastasis (the choroid in 40% of cases and the iris in 2% of cases). MRI is still the preferred diagnostic modality for ocular and extraocular metastasis [9]; in our study, 42% of cases were incidentally detected by MRI and, in 53% of cases, diagnosis and decision for treatment were made radiologically without the need for biopsy. 

Our study identified several predictive factors associated with an increased likelihood of ocular metastasis in breast cancer. In terms of breast cancer subtypes, invasive ductal carcinoma (IDC) and invasive lobular carcinoma (ILC) exhibit differences. While most invasive breast cancers consist of carcinomas of the ductal type, about 10% are invasive lobular carcinomas [26] and ILC is known to have distinct clinical and prognostic features and tends to metastasize to rare organs like the colon and other organs [5,27]. In our cohort, primary breast cancer pathology was IDC in 62% of cases and ILC in 38% of cases, which is higher than the prevalence of lobular carcinoma in the general breast cancer population [26]. Additionally, ocular metastasis occurred in 2.1% of patients with ILC compared to only 0.35% in IDC; therefore, clearly patients with ILC are at significantly much higher risk of developing ocular metastasis than IDC. Furthermore, aligning with prior studies suggesting a distinct metastatic pattern for ILC, this subtype, potentially involving the optic nerve [5], in our study showed that ILC correlated with optic nerve involvement and was also more associated with concomitant brain metastasis. These correlations emphasize the need for tailored approaches in the surveillance and management of breast cancer subtypes, as reported by Heitz et al. [28]. Negative family history of breast cancer and the presence of brain metastasis were also correlated with an elevated risk of ocular involvement, consistent with findings reported by Amer et al. [29].

Advances in diagnostic tools and improved survival for breast cancer patients contribute to the increased occurrence of ocular metastasis. However, recent cases indicate improved treatment outcomes with the use of CDK4/6 inhibitors and stereotactic body radiotherapy (SBRT) [30]. External beam radiotherapy, with a typical dose ranging from 20 to 50 Gy, stabilizes or restores vision in up to 86% of patients [9,11,21,31,32,33].

Management of intraocular metastasis is usually radiation therapy to preserve the eye globe; however, surgery (orbital exenteration or tumor excision) was utilized for orbital metastasis [11]. In our cohort, the primary modality for managing ocular metastasis in breast cancer was radiation therapy (89%), utilizing a standard dose of 30 Gy/10 Fx. Systemic chemotherapy (11%) was employed, particularly in cases with widespread metastatic burden and, at the last follow-up, 55% had excellent visual acuity and 26% had reasonable functional vision. No single case in our study mandated orbital exenteration. Exenteration is usually used for primary orbital tumors like orbital SCC and lacrimal adenoid cystic carcinoma [20]. Complications post-radiation therapy were not negligible, with dry eye disease being the most prevalent, aligning with anatomical considerations of the orbit and choroid, which may contribute to ocular surface abnormalities. Recognition and proactive management of treatment-related complications are crucial for optimizing the quality of life in these patients, in agreement with Shields et al., highlighting the importance of anticipating and managing treatment-related side effects [22]. At the last follow-up, a substantial proportion of patients (71%) with ocular metastatic breast cancer succumbed to the disease. The median duration between ocular metastasis and death was 10 months, emphasizing the aggressive nature of this manifestation, consistent with the findings reported by Gombos et al., who observed a median survival of 12 months in patients with ocular metastasis [34]. While our study provides valuable insights, certain limitations need acknowledgment. The retrospective nature introduces inherent biases and the relatively small sample size limits generalizability. Future research endeavors should aim to expand the cohort size, possibly through multi-center collaborations, to enhance statistical power and refine prognostic assessments. Additionally, investigations into the molecular underpinnings of ocular metastasis, including the role of specific genetic markers, could offer a more nuanced understanding of its pathophysiology.

## 5. Conclusions

In conclusion, our study unravels the clinical intricacies of ocular and periocular metastasis in breast cancer, providing a foundation for informed clinical decision making. Identifying prognostic factors, correlating with primary breast cancer pathology, and assessing therapeutic outcomes contribute to a comprehensive understanding of this rare but impactful manifestation. The findings underscore the need for tailored surveillance strategies, interdisciplinary collaboration, and proactive management of treatment-related complications in this subset of breast cancer patients.

## Figures and Tables

**Table 1:** Demographics and presentation of 45 females with breast cancer who had ocular or periocular metastasis.

Age at Diagnosis of Breast Cancer		Median 48, Mean 48.3, Range 31–71 Years	
Age at Diagnosis of Ocular Metastasis		Median 50, Mean 50.6, Range 35–79 Years	
Breast cancer laterality	Unilateral	39	87%
	Bilateral	6	13%
Pathology of breast cancer *	Invasive ductal	28	62%
	Invasive lobular	17	38%
Family history of breast cancer	Yes	12	27%
	No	33	73%
Site of ocular metastasis	Orbit ^#^	21	47%
	Choroid	18	40%
	Optic nerve	5	11%
	Iris	1	2%
Presenting signs of ocular metastasis	Incidental by MRI (Asymptomatic)	19	42%
	Poor vision	14	31%
	Orbital swelling/Ptosis	5	11%
	Diplopia/Strabismus	4	9%
	Proptosis	3	7%
Method of Diagnosis	Radiological	21	47%
	Clinical	19	42%
	Pathological	5	11%
Laterality	Bilateral	20	44%
	Right or Left	12 / 13	56%
Vision at diagnosis (65 eyes)	less than 0.1	14	22%
	0.1–0.5	19	29%
	More than 0.5	32	49%

* Note: 42 patients we ER + ve (93%), 42 were PR + ve (93%), and 8 patients were HER + ve (18%). ^#^ Three of them had metastasis to extraocular muscles.

**Table 2:** Prognostic factors for ocular and periocular metastasis in patients with breast cancer.

			Number Breast Cancer	Breast Cancer with Ocular Mets	*p* Value
1	Number of breast cancer patients		9902	45 (0.45%)	
2	Pathology	Invasive Ductal	8028	28 (0.35%)	>0.0001
		Invasive Lobular	797	17 (2.1%)	
		NA	1077	0	
3	Age at diagnosis	>50	5148	25 (0.48%)	0.656
		>50	4754	20 (0.4%)	
4	Stage	Stage T1/2	4862	6 (0.12%)	0.0019
		Stage T3/4	1687	9 (0.53%)	
		NA	3351	30 (0.89%)	
5	Distant metastasis	Metastatic	1888	45 (2.4%)	>0.0001
		Nonmetastatic	7644	0 (0%)	
		NA	370	0	
6	Overall Survival	Alive	7924	13 (0.16%)	>0.0001
		Dead	1978	32 (1.6%)	
7	Survival for metastatic breast cancer	Alive	962	13 (1.4%)	0.003
		Dead	926	32	

NA: these data were not available.

**Table 3:** Correlation between the pathology of the breast cancer and the ocular metastasis.

		Invasive Ductal	Invasive LOBULAR	*p* Value
Number		28 (62%)	17 (38%)	
Laterality of ocular metastasis	Unilateral	15 (54%)	10 (59%)	0.48
	Bilateral	13 (46%)	7 (41%)	
Site of Ocular metastasis	Choroid	11 (39%)	7 (41%)	0.62
	Orbit	15 (53%)	6 (35%)	0.17
	Optic nerve	1 (4%)	4 (24%)	0.036
	Iris	1 (4%)	0 (0%)	1.0
Concomitant brain metastasis	Yes	14 (50%)	13 (76%)	0.026
	No	14 (50%)	4 (24%)	
Genetics of Breast Cancer *	Positive	5 (36%)	9 (64%)	0.31
	Negative	4 (57%)	3 (43%)	

* Genetic testing was conducted for BRCA1 and 2 and others.

**Table 4:** Management and outcome of the ocular and periocular metastasis in 45 females with breast cancer.

Treatment of metastasis	Chemotherapy	5	11%
	Radiation	40	89%
Vision after treatment *	Less than 0.1	12	19%
	0.1–0.5	17	26%
	More than 0.5	36	55
Complications	Dry eye	42	93%
	Radiation keratopathy	4	8%
	Radiation retinopathy	3	7%
	Cataract	2	4%
	Periocular redness	6	13%
Survival	Alive	13	29%
	Dead	32	71%
Duration between ocular metastasis to death		Median 10, Mean 16, Range 1–65 months	

* Visual acuity in decibel.

## Data Availability

Data are available on reasonable request on demand to the corresponding authors.
